# Biomechanics-based analysis of technical characteristics in skeleton start and specific physical training strategies

**DOI:** 10.3389/fphys.2025.1700394

**Published:** 2026-01-07

**Authors:** Lei Hao, Qingtao Kong

**Affiliations:** Department of Physical Education and Sport, Shanghai Ocean University, Shanghai, China

**Keywords:** skeleton, starting, technical characteristics, training, winter sports

## Abstract

**Purpose:**

This study employed kinematic and kinetic testing to analyze the sport-specific parameters of the skeleton start, thereby elucidating its technical characteristics and establishing an empirical basis for training.

**Methods:**

Spatiotemporal parameters were captured using a 3D motion system, while ground reaction forces, impulses, and plantar pressure distribution were collected via a Ki-Sprint force platform and custom pressure sensors. Statistical analysis employed descriptive statistics, two-way ANOVA to evaluate factor effects, and appropriate parametric or non-parametric tests for group comparisons.

**Results:**

Athletes’ average starting distance was 18.45 ± 2.09 m, achieved in 12.20 ± 1.11 steps, with no gender difference. Starting speed (male: 6.97 ± 0.42 m/s, female: 6.25 ± 0.58 m/s) and acceleration range (male: 3.31–6.26 m/s^2^, female: 2.14–5.96 m/s^2^) differed significantly by gender. Step length differed between push-off (1.60 ± 0.14 m) and follow-up steps (2.09 ± 0.18 m), and between inner (2.60 ± 0.14 m, 0.44 ± 0.03 s) and outer steps (2.64 ± 0.13 m, 0.45 ± 0.03 s) in both length and duration. Take-off (2.71 ± 0.50 m) and preceding step (2.81 ± 0.58 m) lengths did not differ. The maximum horizontal force was 826.99 ± 217.18 N and average horizontal impulse was 264.43 ± 67.64 N·s, neither correlating with front-rear foot spacing. Average plantar pressure was higher during run-up (1.77 ± 0.76 kg/cm^2^) than take-off (1.19 ± 0.59 kg/cm^2^).

**Conclusion:**

The skeleton start is characterized by periodic speed-power movements akin to a hybrid of sprint acceleration and diving. Training should integrate regular sprints, specialized push simulations, and downhill sprinting to improve acceleration and maximal speed. Core stability, lower-limb maximal strength, and multi-joint power training should be implemented with consideration for gender-specific adaptations.

## 1 Introduction

Originating in Switzerland, skeleton is recognized as the world’s first downhill sliding sport and one of the oldest winter sports (International Olympic Committee, 2018). It is a high-risk activity requiring precise control and imposing rigorous physiological and psychological demands on athletes (Hao et al., 2020). Performed under naturally frigid and physically demanding conditions, the sport embodies a spirit of confrontation against extreme challenges, contributing to its enduring appeal (Hao, 2020; Tian et al., 2022).

The entire process of Skeleton racing can be divided into two stages: the pushing stage and the descending stage. To initiate a Skeleton sled, the athlete first pushes the sled away from the starting block, accelerates it with rapid consecutive pushes while sprinting a distance, and then dives onto the sled in a streamlined posture (International Bobsleigh and Skeleton Federation, 2020).

In the starting phase of skeleton racing, the sled-pushing run-up and the jump onto the sled constitute the sole source of the sled’s kinetic energy. In this phase, athletes leverage human body force generation, the interaction forces between spiked shoes and the ice surface, and other factors to maximize the kinetic energy provided to the sled (Hao et al., 2020; Tian et al., 2022). Also, how effectively their kinetic energy is exerted on the sled is affected by such factors as sports-specific technical movements, pushing-running distance and speed, and other dynamic effects.

Previous studies have covered areas such as athletic performance (Yin et al., 2020), Characteristics of skeleton (Han et al., 2021), physical training in the start phase (Hao, 2020), the physics of ice surfaces (Braghin et al., 2014) and simulation system (Wang et al., 2025). Some research has shown that the performance of the starting stage has an amplifying effect on the performance of the descending stage (Zanoletti et al., 2006). Sled-pushing speed enhancement and sled-landing speed loss reduction are critical to improving skeleton’s start speed (Zanoletti et al., 2006). Colyer (2015) proposed the ‘sled acceleration index’ to assess athletes’ start performance during the pushing phase. The study identified pushing speed, pushing distance, and number of run-up steps as primary correlates of pushing performance. But other studies have indicated that the influence of start speed and start distance on sled-pushing run-up speed and sled-landing effectiveness is relatively independent (Bullock and Martin, 2008). Prior research has examined parameters such as starting speed and distance, though findings remain inconsistent. However, sports biomechanical investigations focusing specifically on the skeleton start phase are scarce, and few studies have derived practical training strategies from a biomechanical perspective. Moreover, existing works often fail to integrate key biomechanical parameters into their analytical frameworks.

Sports biomechanics provides a critical framework for elucidating the underlying mechanisms of skeleton start performance. The accumulation of kinetic energy during the starting phase serves as the intrinsic driving foundation, while the starting speed and starting time represent its extrinsic manifestations (Gong et al., 2023). This study examines the start phase from two complementary perspectives: externally, by analyzing relationships among performance parameters such as distance, speed, and impulse; and internally, by investigating correlations between technical movements and their corresponding kinetic parameters throughout the start sequence.

For start parameters, gender-based differences in start distance and speed were hypothesized. In kinematic analysis, hypotheses centered on sport-specific technical movements, particularly comparing parameters such as medial–lateral or adjacent step lengths. Dynamically, hypotheses were derived by examining similarities and differences in plantar pressure parameters across genders or between consecutive movements. By testing sport-specific techniques and quantifying their mechanical correlates, this study establishes a foundation for deeper investigation into such movements and provides a reference for implementing scientifically informed training.

## 2 Materials and methods

### 2.1 Participants

Twenty athletes from the Chinese National Skeleton Team, who were preparing for the 2022 Beijing Winter Olympics, were selected as participants in this study. All participants had accumulated long-term systematic training experience; among them, some had claimed medals at the International Bobsleigh & Skeleton Federation (IBSF) World Cup and World Championships. Additionally, all athletes were officially registered with the IBSF (International Bobsleigh & Skeleton Federation), meeting the professional qualification criteria for elite skeleton athletes (Table 1).

**TABLE 1:** Basic information of test subjects.

	Height (cm)	Weight (kg)	Age (years)	Training years
*N* = 9 male	180.27 ± 2.83	78.69 ± 4.21	22.11 ± 0.81	5.6 ± 1.4
*N* = 11 female	172.31 ± 5.30	65.53 ± 6.48	19.91 ± 1.55	4.7 ± 1.6

All participants signed an informed consent form before participating in the study. The study was conducted in accordance with the Declaration of Helsinki and approved by the Capital University of Physical Education and Sports Ethics Committee (2020A92).

A *post hoc* power analysis was performed using G*Power 3.1 software to verify the minimum sample size required for the research. The type of statistical test is set as ‘Means: Difference between two independent means, two groups’, with an alpha error (α) set at 0.05. The SD pooled, calculated from the average start speeds of Male (6.9 ± 0.42 m/s) and Female (6.25 ± 0.58 m/s), was approximately 0.52 (SD pooled ≈ 0.52). The effect size, computed via G-Power with the above parameters, was 1.38 (Effect size ≈ 1.38). Further, when the same parameters were re-entered into G-Power, the resulting test power (1-β) was 0.83. This indicates that under the condition that the research hypothesis holds true, the probability of the study correctly detecting this effect is 80%. This constitutes a universally acknowledged ‘acceptable power level’ in statistics, which demonstrates that the study has adequate ‘detecting power’ and possesses explicit practical guiding value.

### 2.2 Experimental protocol

A 3D motion capture system, a plantar pressure sensing system, and the Ki-Sprint sprint start force measurement system were employed to collect and analyze the kinematic and dynamic data of the subjects in this study, aiming to explore the sport-specific physical fitness characteristics. However, as equipment capable of data synchronization has not yet been commercially available, the devices used in this study can only conduct segmented research, and data synchronization cannot be achieved.

#### 2.2.1 Motion capture

Relevant studies on motion capture technology in linear running scenarios provide theoretical and methodological references for the design of data collection protocols and the construction of evaluation indicator systems in this study. Motion capture was employed to measure and evaluate key kinematic parameters, including step length, step frequency, and average speed, during the final 10-m sprint phase of an elite 100-m sprinter Bingtian Su, in a major international competition (Ting-gang and Guo-jie, 2018).

Testing Site: The Skeleton & Bobsleigh pushing track of the National Snow Sports Training Centre in Ankang, Shangxi was used as the test venue in this study. The start training track was reproduced in equal proportion based on the construction parameters of the skeleton race track at the National Sliding Centre (Yanqing). In terms of track shape, gradient, and timing system, its construction specifications were consistent with those of the start section of the skeleton race track used in the 2022 Beijing Winter Olympics.

The starting phase of the skeleton track commences at the starting pedals and is equipped with two ice grooves (17 mm in diameter) that match the runners of the skeleton sled. The descending gradient of the track section from 0 to 15 m is 2%, while that of the section from 15 to 75 m is 12%. The specifications for the starting phase are consistent across all tracks (FIBT, 2022).

Testing Equipment: Fastmove-3D motion capture equipment and an analysis system were used to perform the motion capture test. This equipment consists of six fast cameras, one QF-26 three-dimensional stereo shooting calibration frame, computers, three-dimensional motion analysis software, etc.

**FIGURE 1:**
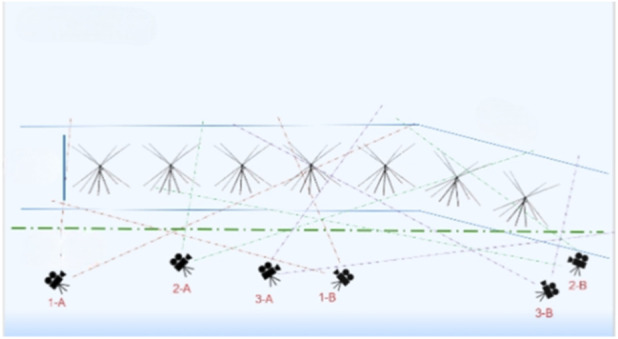
Camera set-up scheme for motion capture.

Camera Position: The entire video capture area is divided into three parts and covers the entire start process of Skeleton athletes. A total of six cameras were installed in these areas, with every two cameras grouped as one set with an angle of 60°–120° were set in each area, for recording data during the motion capture process. Meanwhile, there was an overlap between every two areas to ensure that the whole track was covered by cameras, constructing a calibration frame for data splicing (Figure 1).

Calibration Protocol: A radial 3D calibration frame was employed to conduct 3D spatial calibration for each segment. Subsequently, the intermediate segment calibration was leveraged to solve for the remaining segment calibrations, and the segmented capture data were stitched based on the principle of deriving global error by comparing segment-specific minimum errors. This approach ensured high calibration accuracy could be maintained across the full movement range in large-scale capture scenarios.

The underlying principle operates as follows: Fundamental spatial relationships among multiple calibration frames are estimated using a single central frame (located at the geometric center of the capture field). Using the minimum reconstruction error as the optimization criterion, the optimal solution for the spatial relationships between multiple frames is derived. Leveraging these optimized spatial relationships, a multi-frame calibration model is established, ultimately yielding a merged calibration model integrating all frame sets. This method stitches 3D motion capture data from three small-scale segments into a continuous data set representing the full movement process, thereby overcoming the inherent limitations of 3D motion capture technology in terms of spatial coverage and segmental discontinuity.

**FIGURE 2:**
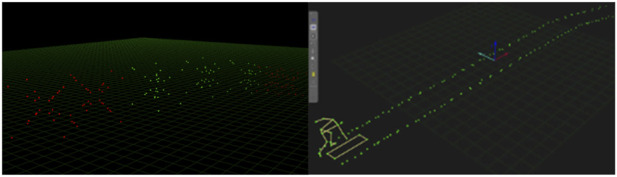
Large-scale space stitching, calibration scheme, and motion analysis images.

Video and Analyzed Data Processing: Adobe Premiere video editing software was used to intercept images of motion capture. The Fastmove-3D-Motion system includes artificial intelligence tie-in motion capture system can measure 21 key points of a human body automatically, and the analysis software has such functions as automatic point supplementation, filtering, and smoothing. It combined with spatial calibration data was used to analyze and supplement the point optimization of two or more identical pictures. With each segmented video analyzed, the motion capture data were spliced to synthesize the motion trajectory, following the steps of time alignment, optimization time alignment, translation correction, rotation correction, translation optimization, and rotation to stability. Based on the Zatsiorlky Human Body Calculation Model, the position and displacement curve of the gravity center were calculated. C# language programming software (Microsoft Visual Studio 2010) was used to calculate data. Figure 2 illustrates the large-scale 3D space stitching, calibration scheme, and 3D motion analysis images.

#### 2.2.2 Kinetic test

Studies on plantar pressure provide a reference basis for the testing in this study. Meanwhile, plantar pressure testing can effectively reflect differences in training levels through pressure values and impulse (Jinxian and Bin, 2014). While Skeleton athletes exert forces on the start pedals and track ice surface respectively during the start phase, the plantar pressure characteristics during this process remain unknown. Therefore, this study will collect and analyze plantar pressure parameters, with the aim of providing a basis for subsequent research.

Dynamic test of sled pushing-off.

Testing Site: Indoor Track and Field Stadium.

Testing Equipment: A Ki-Sprint starting multi-component pressure measurement system and Skeleton simulation training device.

**FIGURE 3:**
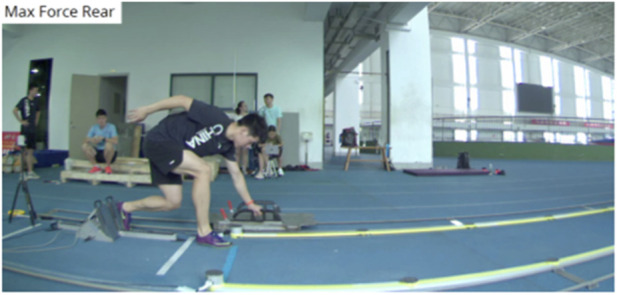
Optimal generating force testing during the push-off stage.

Testing Process: Before the test, the pressure-measuring pedal was adjusted to the highest angle of 70° and was placed in front of the push sled and simulated track. Test subjects placed their front feet in a fixed position. Taking the full length of each subject’s calf as the starting distance, the pressure measuring pedal was moved back in the 3.5 cm for 2nd to 5th tests. Based on the corresponding distance between the front and rear feet of a subject, data of horizontal maximum force and horizontal impulse were measured (Figure 3).

Dynamic test of running-off and taking-off.

Testing Site: National Snow Sports Training Centre, Ankang.

**FIGURE 4:**
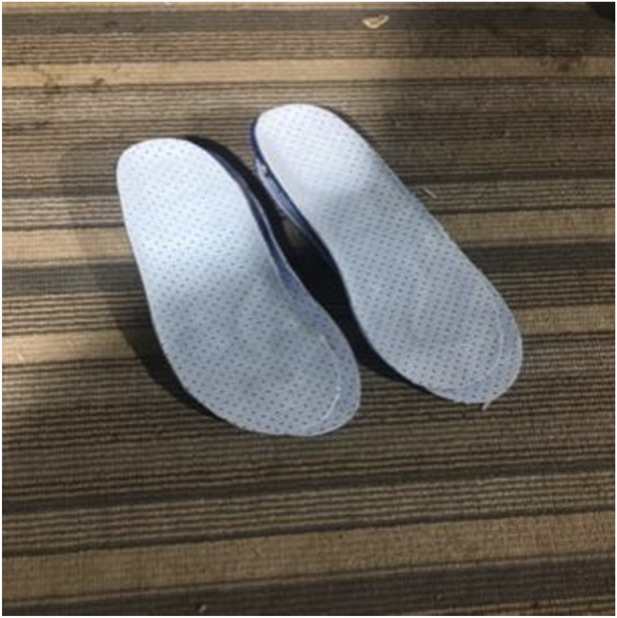
The customized foot-pressure measurement instrument.

Testing Equipment: The customized foot-pressure measurement instrument that was invented by the Institute of Microelectronics, Chinese Academy of Sciences. Three pressure sensors and one acceleration sensor were placed in each test instrument (Figure 4).

**FIGURE 5:**
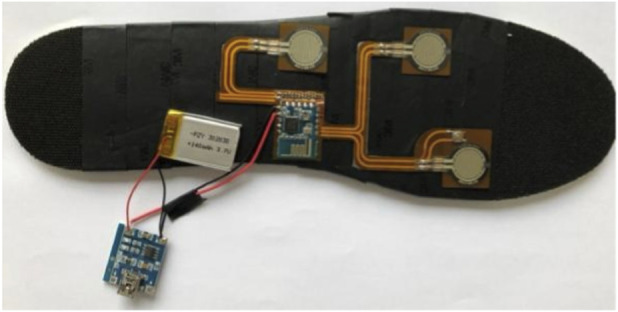
The physical map of smart wearable device.

**FIGURE 6:**
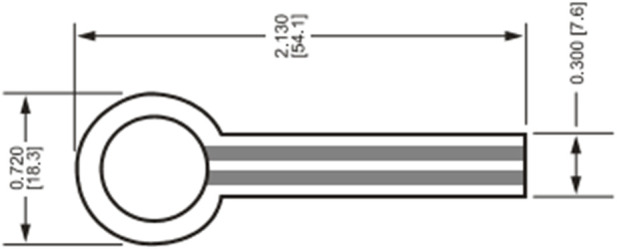
The schematic of FSR402.

The thin-film pressure sensor consisting of polyester film with excellent performance and high-conductivity materials and nano-scale pressure-sensitive materials is used in the instrument. The piezoresistive thin-film pressure sensor FSR402 (Produced by InterLink Electronics) with good flexibility is selected to measure the pressure distribution of the human plantar. The diameter of its effective area is 12.7 mm, the thickness is 0.46 mm, the measuring range is 0–10 kg, the resolution is 50 g, the measurement accuracy is ±2%–±5%, and the maximum operating current does not exceed 1 mA (Figures 5, 6).

**FIGURE 7:**
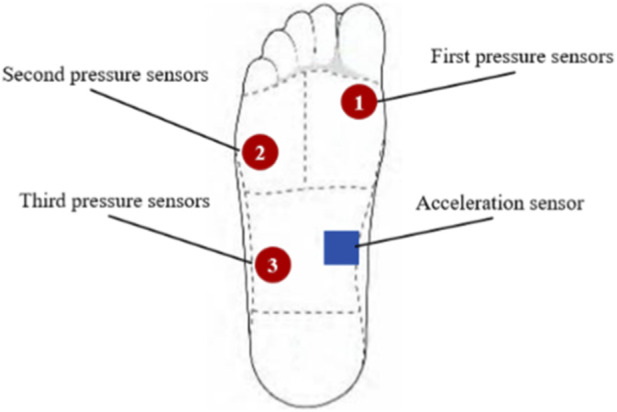
The place of pressure sensors and acceleration sensor.

Based on the research findings of plantar pressure distribution among 158 subjects (Yuan et al., 2004). And the actual situation where the heel of Skeleton athletes does not touch the ice surface during the start. The pressure sensors were placed on the first metatarsal, the fifth metatarsal, and the outer side of the arch. The pressure-weight ratios of the first metatarsal bone, the fifth metatarsal bone, the outer arch and the heel of every athlete were set as 50%, 35%, 15% and 0%, respectively (Figure 7).

The acceleration sensor was placed on the inner side of the arch. Data were transferred through the Bluetooth 5.0 receiver and transmitter fixed on the computer and sled, respectively.

#### 2.2.3 Experimental control and trials repetition

Familiarization Trials: To mitigate practice-induced confounding, three familiarization trials (non-data recorded) were performed in advance of the formal test, allowing participants to gain mastery of starting rhythm and technique.

Formal Retest: The parameters for speed capture primarily included average speed, maximum speed, and acceleration. For spatial capture, the focus was placed on distance covered, step length, and step frequency. The primary parameter collected during kinetic testing was the plantar pressure of each participant.

Based on the test intensity and fatigue sensitivity, with a 10-min rest interval between trials. This interval was implemented to ensure that the participants’ heart rate returned to within 10% of the baseline level, thereby preventing the accumulation of anaerobic metabolites from interfering with subsequent trials.

The tests were conducted in the same space and within the same time period. On the day of the test, the ice temperature of the track ranged from −3 °C to −5 °C, and the ice surface was renovated once after every five participant-tests were completed.

### 2.3 Statistical analysis

Microsoft Office Excel and SPSS 22.0 were used to organize and analyze the data involved in this study. Descriptive statistical analysis is presented using mean ± SD. The normal distribution data were first tested, and then with the two-way analysis of variance (ANOVA), T-test, and non-parametric tests were conducted on the kinematics and dynamics data, with a *p* < 0.05 indicating a statistically significant result.

## 3 Result

### 3.1 Motion capture of skeleton start’s distance and speed

Based on the displacement distance of gravity center of each athlete’s starting Skeleton, the start distance and start steps of the athlete were calibrated, with his or her moving speed and acceleration calculated.

**FIGURE 8:**
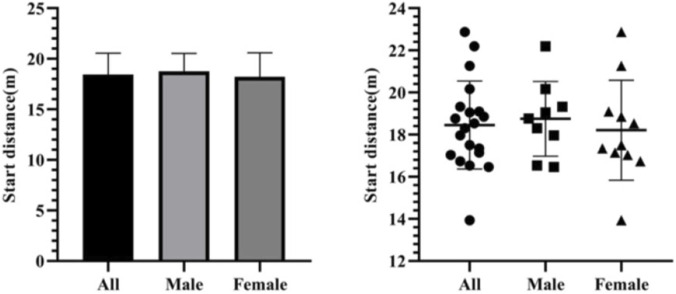
Start distances of Chinese skeleton athletes.

**FIGURE 9:**
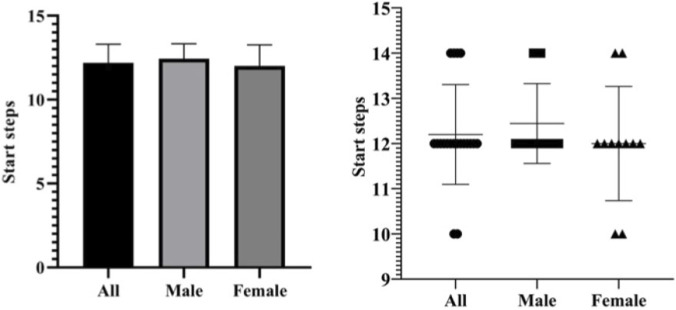
Start steps of Chinese skeleton athletes.

Among all test subjects, the start distances ranged from 13.94 to 22.87 m, with an average distance of 18.45 ± 2.09 m. The start steps of all test subjects ranged from 10 to 14 steps, with an average of 12.20 ± 1.11 steps. Among male athletes, the start distances ranged from 16.46 to 22.20 m, with an average distance of 18.75 ± 1.78 m. And their start steps ranged from 12 to 14 steps, with an average of 14.44 ± 0.88 steps. Among female athletes, the start distances ranged from 13.94 to 22.87 m, with an average distance of 18.21 ± 2.38 m. And their start steps ranged from 10 to 14 steps, with an average of 12.00 ± 1.26 steps (Figures 8, 9).

**FIGURE 10:**
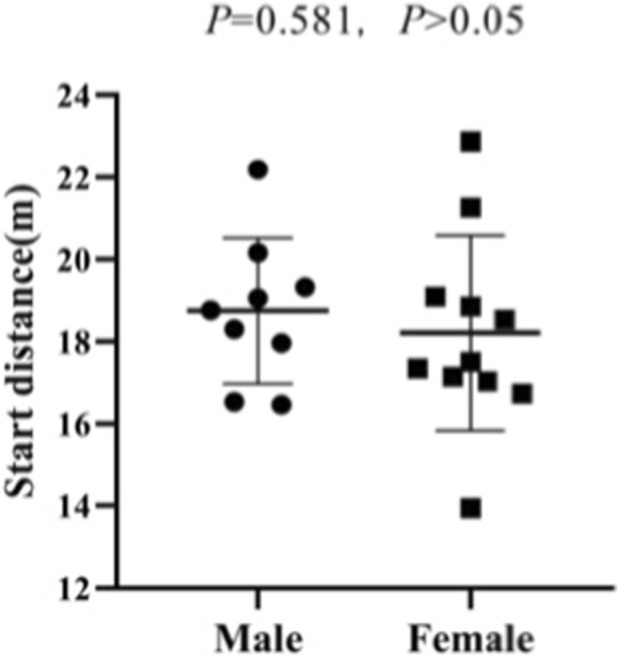
Start distance results of the independent sample t-test on male and female athletes.

The start distance data follow a normal distribution (Shapiro-Wilk test *p* = 0.585). The independent sample T-test shows that there is no significant difference between the start distances of male and female athletes (*p* = 0.581, *p* > 0.05) (Figure 10).

**FIGURE 11:**
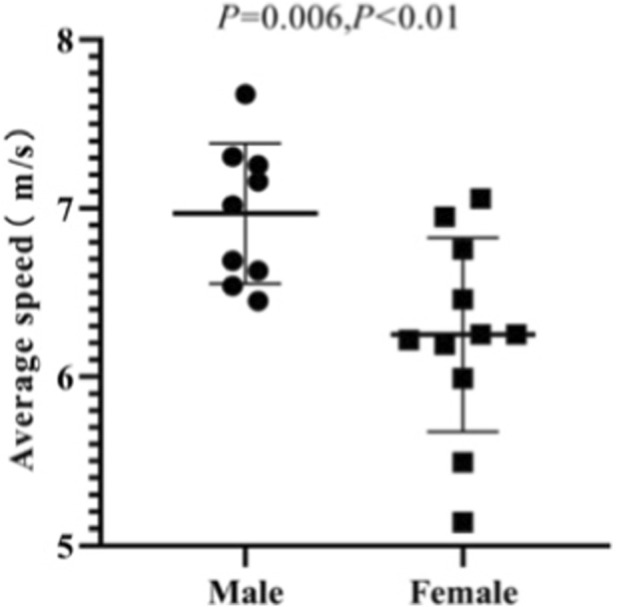
Average start speeds of male and female athletes.

**FIGURE 12:**
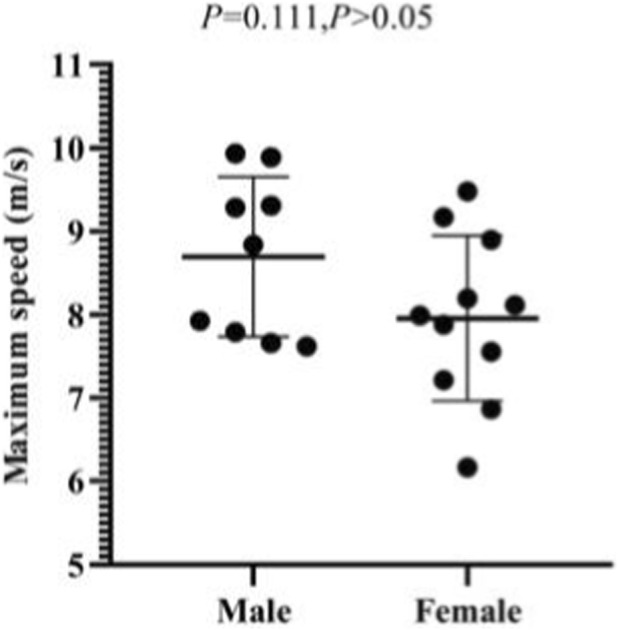
Maximum start speeds of male and female athletes.

**FIGURE 13:**
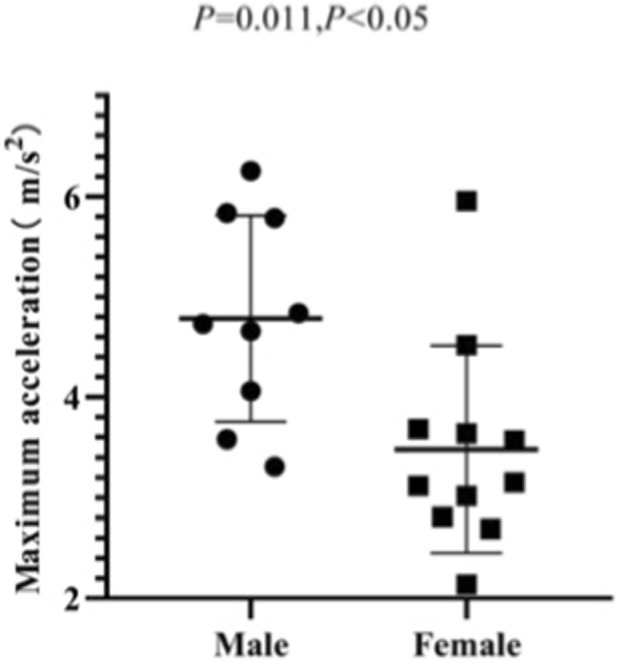
Maximum start acceleration of male and female athletes.

Among male athletes, the average start speed is 6.97 ± 0.42 m/s, the maximum start speed ranges from 7.62 to 9.94 m/s, and the acceleration ranges from 3.31 to 6.26 m/s^2^. Among female athletes, the average start speed is 6.25 ± 0.58 m/s, the maximum start speed ranges from 6.16 to 9.48 m/s, and the acceleration ranges from 2.14 to 5.96 m/s^2^. The results of the independent sample T-test show that there is no significant difference between the maximum start speeds of male and female athletes (*p* = 0.111, *p* > 0.05). However, there are significant differences between the average start speeds (*p* = 0.006, *p* < 0.05) and maximum acceleration (*p* = 0.011, *p* < 0.05) of male and female athletes (Figures 11–13).

It can be concluded that male and female athletes can achieve similar maximum start speeds. However, there are significant differences between the average start speeds and maximum acceleration of male and female athletes, with the average start speed and maximum acceleration of male athletes being stronger than those of female athletes.

### 3.2 Skeleton start gait kinematics test of Chinese athletes

The Skeleton start track is a downhill ice surface with a 2% slope. Athletes training in this start track have asymmetric pushing step lengths and stride frequencies which are different from those of athletes training on a flat ground track (Luo, 2012; Huo and Xu, 2009). In a good sprint technique, stride length and stride frequency are the main factors determining running speed.

#### 3.2.1 Push-off and follow-up step length kinematics test of Chinese skeleton athletes

**FIGURE 14:**
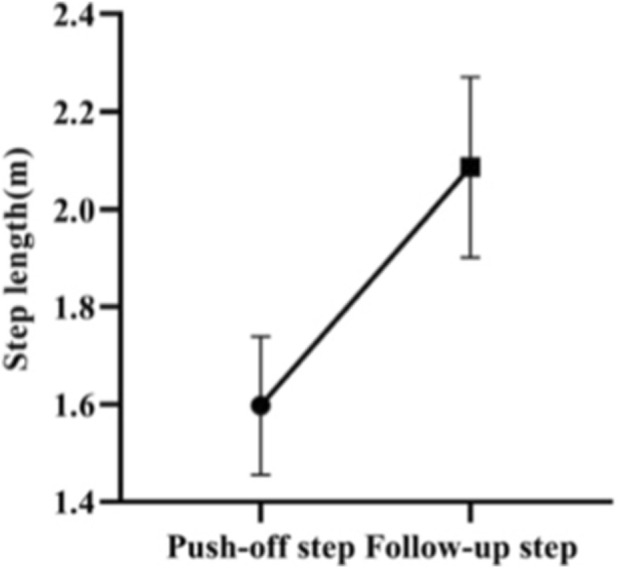
Push-off step length and follow-up step length.

**FIGURE 15:**
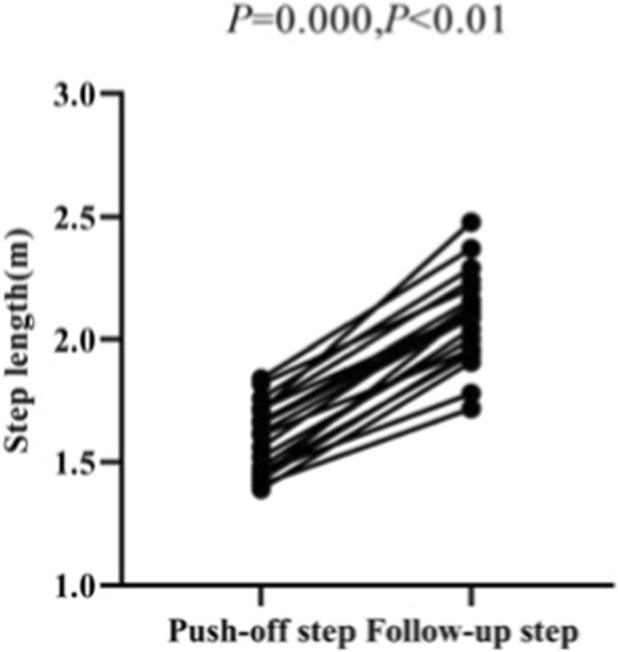
Push-off step length and follow-up step length measured in the paired samples t-test.

In this study, the average push-off step length of the test subjects during the Skeleton start is 1.60 ± 0.14 m, and the average follow-up step length of the test subjects during the Skeleton start is 2.09 ± 0.18 m. The paired sample t-test shows that there is a significant difference between the push-off step lengths and the follow-up step lengths of Skeleton athletes (*p* = 0.000, *p* < 0.01), with their follow-up step lengths being longer than their push-off step lengths (Figures 14, 15). It can be concluded that the follow-up step lengths of Skeleton athletes are longer than their push-off step lengths during the Skeleton start. That is, after pushing off the pedal, a Skeleton athlete needs to take a long stride to move forward.

#### 3.2.2 Measured running-off step length and frequency in the kinematics test

The running-off gait of Skeleton start is similar to the gait of a sprint. A mid-run stage consists of two supporting periods and two flying periods. The supporting period begins with one foot hitting the ground and ends with the other foot lifting off the ground, while the flying period begins with one foot lifting off the ground and ends with the other foot touching the ground. It can be concluded that the step lengths and step frequencies of both feet are asymmetrical. That is, the length of the inside step is longer than the length of the outside step, and the frequency of the inside step is higher than the frequency of the outside step during the sled running-off period. Based on the step length defined by running technique, the lengths and frequencies of the inside and outside steps can be calculated.

**FIGURE 16:**
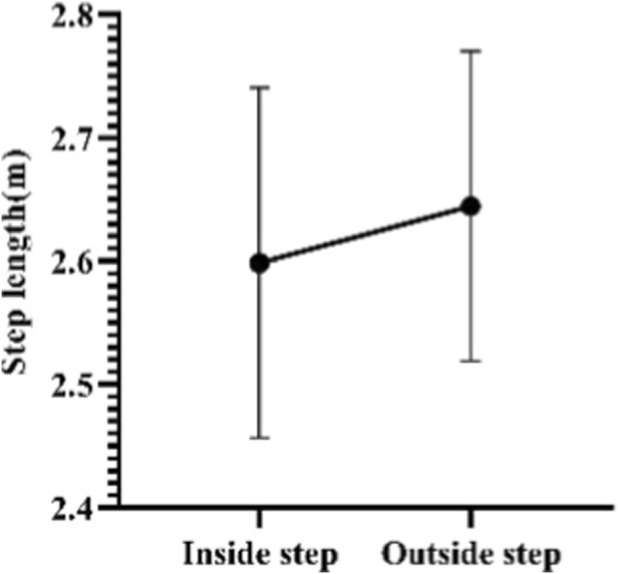
Lengths of Skeleton athletes’ inside and outside steps during the sled running-off period.

**FIGURE 17:**
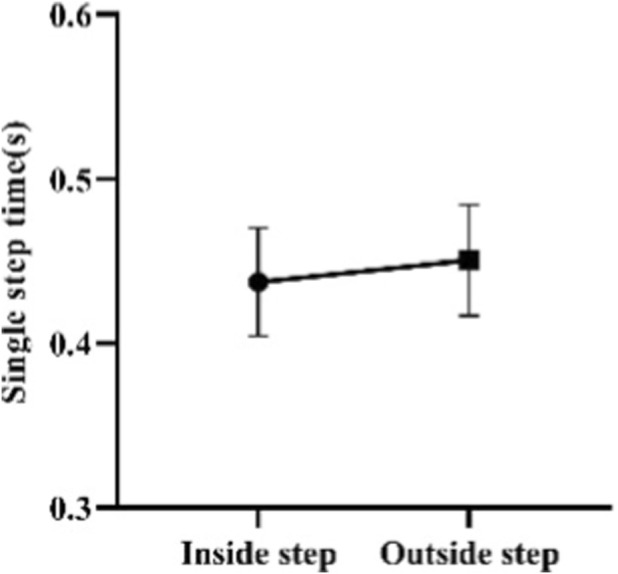
Single-step time of Skeleton athletes’ inside and outside steps during the sled running-off period.

In this study, the step length and frequency data during the sled running-off period (excluding the pushing-off and landing periods) were measured. The lengths of the test subjects’ inside steps during the sled running-off period range from 2.33 to 2.85 m, with an average length of 2.60 ± 0.14 m. And the lengths of their outside steps during the sled running-off period range from 2.34 to 2.87 m, with an average length of 2.64 ± 0.13 m. Meanwhile, the time of their inside steps ranges from 0.39 to 0.52 s, with an average of 0.44 ± 0.03 s. And the time of their outside steps ranges from 0.40 to 0.53 s, with an average of 0.45 ± 0.03 s (Figures 16, 17).

**FIGURE 18:**
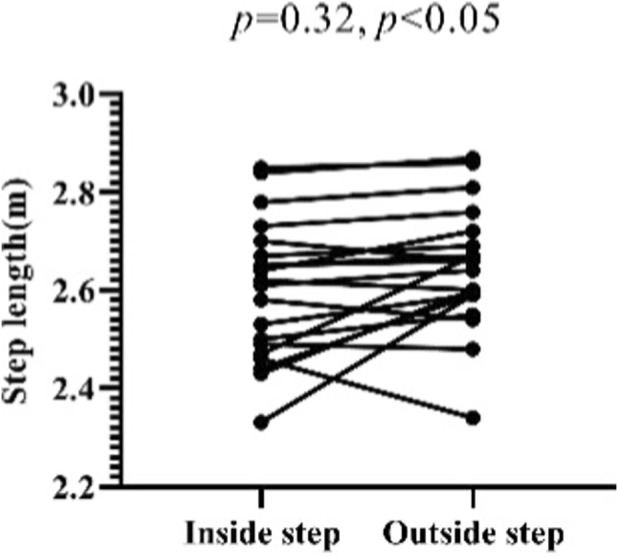
Inside and outside step lengths of Skeleton athletes measured in the t-test.

**FIGURE 19:**
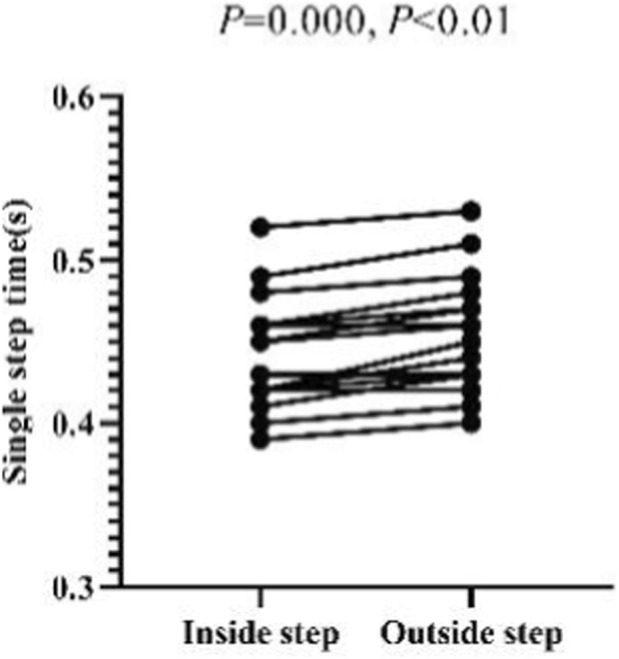
Single-step time of inside and outside steps of Skeleton athletes measured in the t-test.

The paired sample t-test shows that there is a significant difference between the lengths of the inside and outside steps of Skeleton athletes during the sled running-off period (*p* = 0.032, 0.01 < *p <* 0.05), and that there is a significant difference between the time of their inside and outside steps during the sled running-off period (*p =* 0.000, *p <* 0.01) (Figures 18, 19). It can be concluded that a Skeleton athlete performs an asymmetrical periodic running movement during the sled running-off period. During this period, the athlete holds the sled with one arm, thus limiting the movement range of his or her ipsilateral lower limb. Therefore, the step lengths and time of his or her feet are asymmetrical.

#### 3.2.3 Kinematics test of take-off and preceding step lengths

Landing on sled is the last step of Skeleton start. The athlete needs to perform a diving jump movement to land on a sled.

**FIGURE 20:**
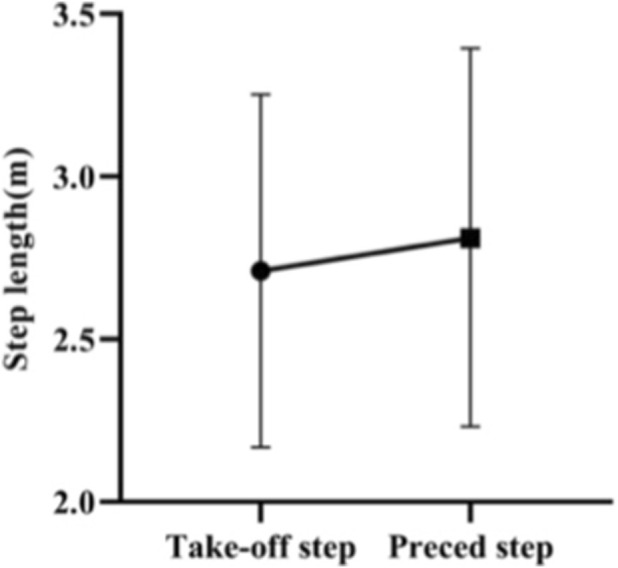
Take-off and preceding step lengths of Skeleton athlete.

**FIGURE 21:**
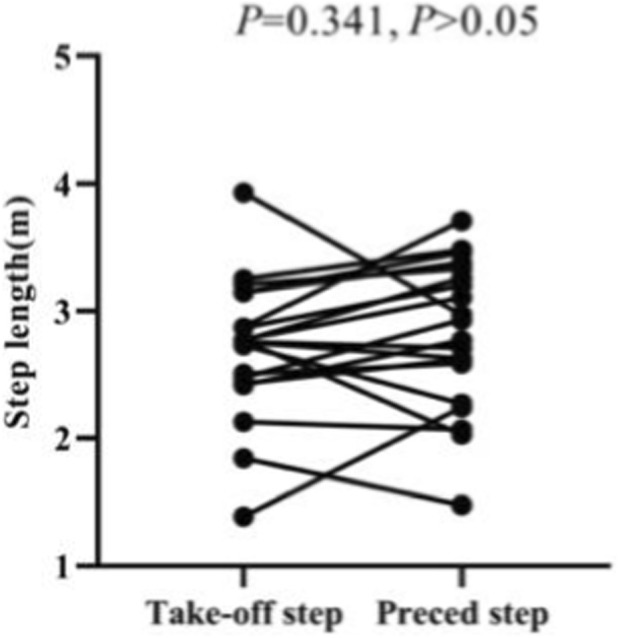
Take-off and preceding step lengths of Skeleton athletes measured in the paired sample t-test.

In this study, a take-off step length of 2.71 ± 0.5 m and a preceding step length of 2.81 ± 0.58 m were measured. Also, the paired sample t-test shows that there is no significant difference between the take-off and preceding step lengths of Skeleton athlete landing on a sled (*p* = 0.341, *p >* 0.05) (Figures 20, 21).

It can be concluded that the take-off and preceding step lengths are similar, and it is not necessary for the athlete to take a longer stride to jump onto the sled than his or her preceding step.

### 3.3 Dynamic test of skeleton start of Chinese athletes

No previous studies have ever investigated such mechanisms as whether the distance between the front and rear feet of a Skeleton athlete will influence the sled pushing-off performance, whether male and female athletes have different plantar force during the running-off period, and whether the hopping feet of athletes need to step on the ground when they jump onto the sled. In this study, a dynamic test was conducted to investigate these mechanisms.

#### 3.3.1 Dynamic test of sled pushing-off

In this study, it is assumed that the distance between the front and rear feet of a Skeleton athlete can affect his or her starting efficiency and such dynamic parameters as horizontal force and horizontal impulse during the sled pushing-off period. Also, with these dynamic parameters, this study has analyzed the mechanical characteristics of the athlete and explored the relationship between the optimal distance between an athlete’s front and rear feet and his or her maximum horizontal force and horizontal impulse.

With a reference to the dynamic approach of sprint start, in this study, five starts were performed to measure such data as maximum horizontal force [Max F-horizontal (N) & (BW)], horizontal impulse [Max I-horizontal (Ns)], and corresponding distances between the front and rear feet of athletes during the sled pushing-off period.

**FIGURE 22:**
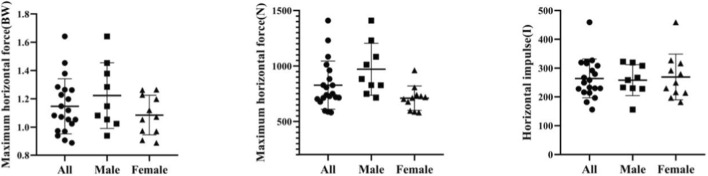
Maximum horizontal force and horizontal impulse.

A mean value of 1.15 ± 0.20 BW & 826.99 ± 217.18 N was obtained on the maximum horizontal force, with a maximum value of 264.43 ± 67.64 Ns obtained on the horizontal impulse. Among male athletes, the mean value of maximum horizontal force is 1.240.23 BW & 985.53 ± 228.26 N, and the mean value of maximum horizontal impulse is 284.04 ± 84.25 Ns. Among female athletes, the mean value of maximum horizontal force is 1.070.13 BW & 697.27 ± 82.52 N, and the mean value of maximum horizontal impulse is 248.37 ± 48.83 Ns (Figure 22).

**FIGURE 23:**
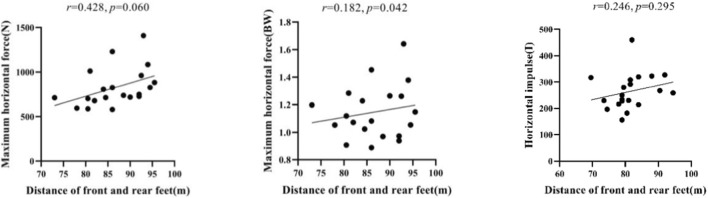
Correlations of the maximum horizontal force and horizontal impulse with the distance between the front and rear feet of an athlete during the sled pushing-off stage.

In this study, a Pearson correlation analysis was carried out, with the result that the maximum horizontal force and the horizontal impulse are not correlated with the distance between the front and rear feet of an athlete (*r* = 0.428, *p* = 0.060, *p >* 0.05; *r* = 0.182, *p* = 0.442, *p >* 0.05; and *r* = 0.246, *p* = 0.295, *p >* 0.05). It can be inferred that the placement distance of the front and rear feet of an athlete during the sled pushing-off stage has no influence on the horizontal force and horizontal impulse of the athlete (Figure 23).

**FIGURE 24:**
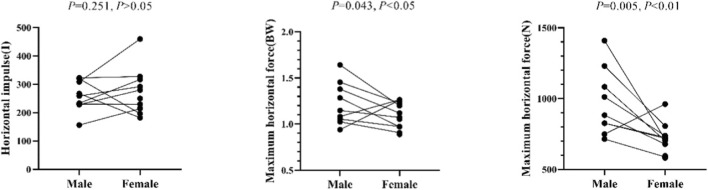
Maximum horizontal force and horizontal impulse of male and female athletes measured in the independent sample T-test.

The independent sample T-test shows that there is no significant difference between the horizontal impulse of male and female athletes (*p* = 0.251, *p >* 0.05), there is a significant difference between the relative maximum horizontal force of male and female athletes (*p* = 0.043, *p <* 0.05) and there are significant differences between the absolute maximum horizontal force and body weights of male and female athletes (*p* = 0.005, *p <* 0.05) (Figure 24).

Based on the following formula, it can be seen that Skeleton athletes with different body weights and strength have different absolute maximum horizontal force during the sled pushing-off stage: F = relative value × body weight × 9.8. Male and female Skeleton athletes have different relative maximum horizontal force, with the force of male athletes greater than the force of female athletes. It can be concluded that the starting advantage achieved by an athlete is related to the maximum strength and explosive power of his or her lower extremities. An athlete with a greater maximum strength of lower extremities will have greater horizontal force and horizontal impulse during the sled pushing-off stage. However, the horizontal force and horizontal impulse of an athlete are not correlated with the corresponding distance between his or her front and real feet. Because male athletes have a greater maximum strength of lower extremities than female athletes, their horizontal force during the sled pushing-off stage is higher than that force of female athletes.

#### 3.3.2 Dynamic test of athlete running-off and taking-off

The running-off movement of an athlete consists of the action of hip flexion, one-side sled holding, one-side arm swing, alternated leg swing and pushing. The jumping-onto-sled action can be viewed as one-leg diving and jumping onto sled movement.

It is assumed that when jumping onto a sled, the athlete will extend his or her take-off leg fully. That is, the plantar pressure of an athlete jumping onto a sled is higher than that pressure of the athlete running off the sled.

**FIGURE 25:**
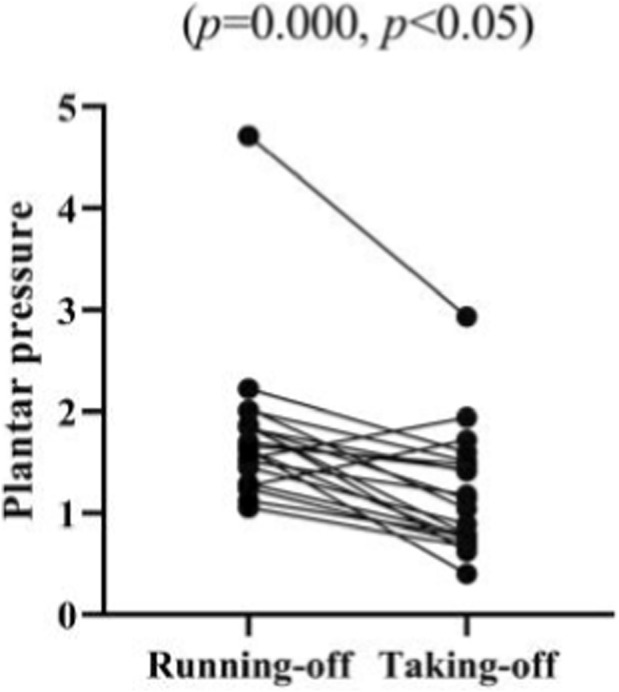
Plantar pressure of athletes during the running-off and taking-off stages.

**FIGURE 26:**
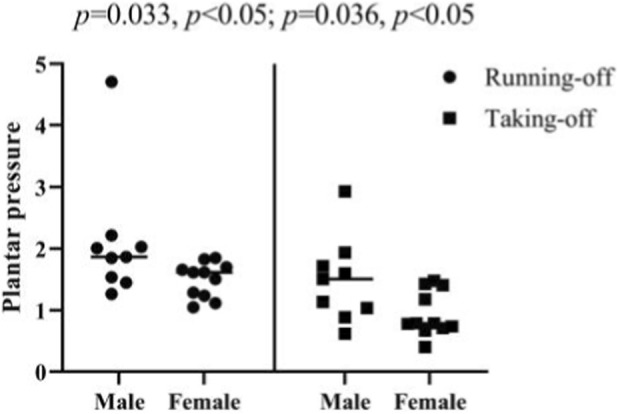
Plantar pressure of athletes during running-off and taking-off stages measured in the paired sample T-test.

The plantar pressure data of athletes during the running-off and taking-off stages were tested and analyzed in this study. The mean value of all test subjects’ plantar pressure during the running-up stage is 1.77 ± 0.76 kg/cm^2^, with the mean values of all male and female participants’ plantar pressure being 2.11 ± 1.02 kg/cm^2^ and 1.50 ± 0.28 kg/cm^2^, respectively. Meanwhile, the mean value of all test subjects’ plantar pressure during the take-off stage is 1.19 ± 0.59 kg/cm^2^, with the mean values of male and female participants’ plantar pressure being 1.49 ± 0.69 kg/cm^2^ and 0.94 ± 0.37 kg/cm^2^, respectively (Figure 25). The paired sample T-test shows that there is a significant difference between the plantar pressure of all test subjects during the running-off and take-off stages (*p* = 0.000, *p <* 0.05), and that there are significant differences between the plantar pressure of male and female participants during these two stages (*p* = 0.033, *p <* 0.05; *p* = 0.036, *p <* 0.05) (Figure 26).

**FIGURE 27:**
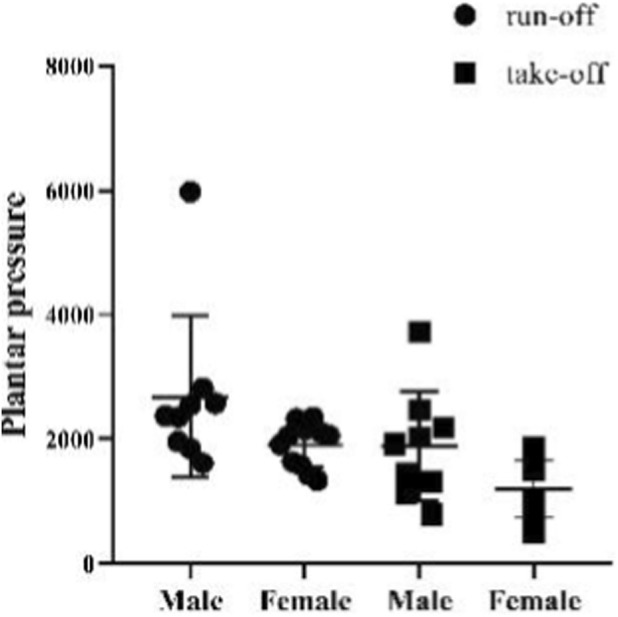
Plantar pressure of male and female athletes during the running-off and take-off periods.

**FIGURE 28:**
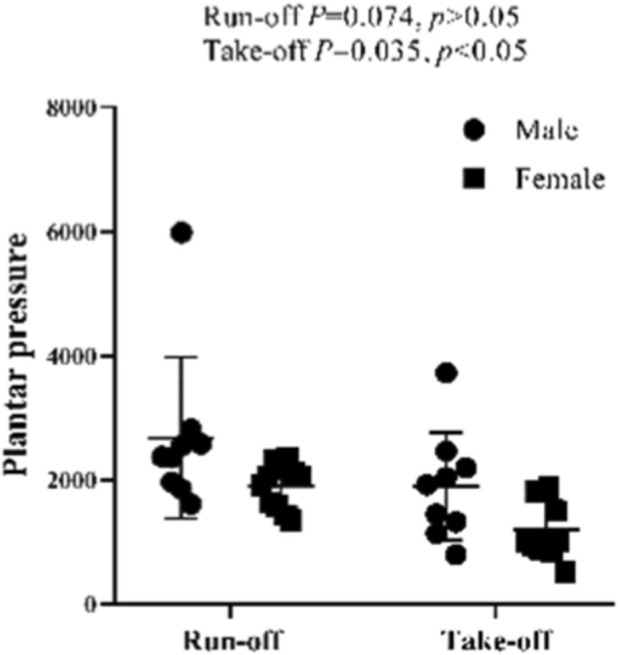
Plantar pressure of male and female athletes during the running-off and take-off periods measured in the independent sample T-test.

The independent sample T-test shows that there is no significant difference between the plantar pressure of male and female athletes during the sled running-off period (*p* = 0.074, *p* > 0.05). However, this test also indicates that there is a significant difference between the plantar pressure of male and female athletes during the take-off period (*p* = 0.035, *p* < 0.05), with the plantar pressure of male athletes significantly higher than that pressure of female athletes (Figures 27, 28).

With the test results, it can be concluded that the ice reaction force during the take-off stage is smaller than that ice force during the running-off stage. Also, there is no difference in the reaction force of ice with running-up between males and females, but the reaction force of males is greater than females in take-off.

## 4 Discussion

### 4.1 Characteristics of start distance and speed of skeleton start and their training implications

The starting tracks for bobsleigh and skeleton competitions adhere to standardized specifications across the globe. Notably, the competition regulations for skeleton do not impose restrictions on the starting distance; this allows athletes to independently determine the number of steps taken and the specific starting distance based on their individual physical fitness levels and pre-competition tactical strategies.

Starting speed serves as an external manifestation of starting motor performance. The interrelationships among starting distance, starting step count, and starting speed warrant further investigation. The results of this study demonstrate that neither athlete gender nor physical capability exerts a significant effect on the starting distance during Skeleton starts. In contrast, both factors significantly influence the average speed and maximum acceleration of athletes in the start phase, with male athletes exhibiting superior average speed and maximum acceleration relative to their female counterparts. However, some studies have drawn opposite conclusions. For instance, Colyer (2015) demonstrated that a long-distance sled push-off enables athletes to achieve higher speeds than short or medium distance counterparts. Furthermore, Colyer et al. (2018) found that each 1-m increase in sled push-off distance is associated with an approximate 0.11 m/s rise in an athlete’s starting speed. From the above research conclusions, it can be inferred that athletes with longer starting distances and more push-off steps are more likely to achieve higher maximum starting speeds and accumulate greater kinetic energy.

Studies conducted in Lake Placid (focusing on tracks modeled after Sigulda and St. Moritz), Colyer et al. (2018) observed that athletes achieved start speeds of 11.44 ± 0.21 m/s, 11.27 ± 0.18 m/s, and 10.97 ± 0.43 m/s when using 18 ± 1 steps, 17 ± 2 steps, and 14 ± 1 steps, respectively. Colyer et al. (2018) noted that the optimal start distances were 28.60 ± 2.68 m and 25.63 ± 1.76 m, with corresponding start steps of 17.4 ± 1.3 and 15.9 ± 1.2 steps. The aforementioned studies indicate that no linear relationship exists between an athlete’s starting speed and either their starting distance or starting step count. Nicola Bullock conducted a study on elite female athletes worldwide and found that their start distances and step counts varied. She further concluded that start distance is influenced by factors such as physical conditions, psychological states, and other relevant variables (Bullock and Martin, 2008).

Through logical reasoning, it can be concluded that: theoretically, extending the distance of the run-off phase to a certain extent contributes to the improvement of Skeleton athletes’ start speed. In practical scenarios, however, athletes with different start capabilities demonstrate variations in three key aspects during the sled run-off phase: start speed, step frequency, and run-off distance. Specifically, the sled run-off speed is primarily influenced by athletes’ physical fitness, with their speed capacity being a particularly critical determinant.

This finding is further corroborated by empirical evidence from specialized research: Colyer’s et al. (2017) study, conducted on a sample of 12 British Skeleton athletes, explicitly identified muscle contraction velocity as the core determinant of Skeleton athletes’ start performance, thereby offering a more nuanced physiological account regarding the association between physical fitness and start speed. Consistent with the aforementioned physiological mechanism, the specific pathway underlying speed improvement is explained as follows: Lower-level and higher-level Skeleton athletes enhance their running speed primarily by extending step length and elevating step frequency, respectively, as demonstrated by Weyand et al. (2000). For Skeleton start performance specifically, key indicators of lower limb explosive power (e.g., peak horizontal ground reaction force during push-off and vertical jump height) and core stability are found to exhibit a significant positive correlation with start performance. This correlation is further quantified by empirical data: Sands and Smith (2005) observed that for every 0.1-s reduction in start time, the lower limb push-off force needs to be increased by approximately 5%–8%.

Consequently, enhancing lower limb explosive power and core stability serves as the fundamental physical fitness prerequisite for improving skeleton start speed. Conversely, athletes with insufficient physical fitness are compelled to mount the sled earlier to prevent a decline in sled velocity, thereby shortening their run-off distance and reducing step count. As a direct result, such athletes fail to continuously accumulate horizontal impulse, which in turn diminishes their start speed. Nevertheless, athletes with limited physical capacity may adopt sled run-off strategies adapted to the track’s first curve configuration. Notably, physical capacities supporting rapid acceleration and sustained maximum speed in athletes constitute key determinants influencing skeleton start performance.

It is therefore recommended that athletes adjust their sled run-off distances in accordance with the track’s first curve configuration, and enhance their acceleration capacity and maximum speed by leveraging the longest feasible run-off distances. Furthermore, athletes should optimize core run-off parameters, including run-off step count, acceleration rate, maximum speed, and speed maintenance duration, through standardized training centered on step placement accuracy and rhythm control.

### 4.2 Technical characteristics of skeleton start and their training implications

**FIGURE 29:**
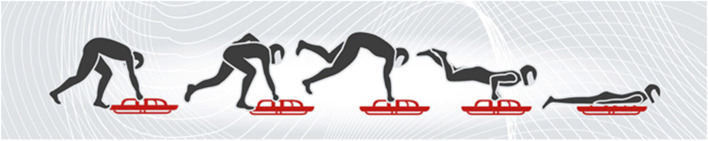
Two stages of Skeleton start (extracted from the IBSF website).

Skeleton start can be divided into two stages: sled pushing-off and sled take-off. Meanwhile, the sled push-off stage can be further subdivided into five sequential phases: sled preparation, driving, transition, maximum speed attainment, and speed sustaining (Figure 29).

#### 4.2.1 Characteristics of athlete preparation position and their training implications

A high-quality start performance enables skeleton athletes to concurrently achieve enhanced impulse, increased initial velocity, and optimized speed rhythm (Mero et al., 2006). The push-off technique for the Skeleton start requires athletes to adopt a squat preparatory posture, with their hip joint flexed and torso in an upright position. This movement consists of the extension of the hip, knee, and ankle joints, and relies on the reaction force from the starting pedal to propel themselves forward.

The experimental results of this study demonstrate that anteroposterior foot spacing confers no significant biomechanical advantage to athletes during sled push-off from a squat preparatory posture. In contrast, mediolateral foot spacing (i.e., left-to-right spacing) should be matched to the athlete’s hip width: an excessively wide stance results in misalignment in the direction of force application. Additionally, the athlete’s hip position exhibits a significant correlation with the knee joint angle. With respect to sled grip, athletes employ a one-handed grip technique, which specifically includes grasping the posterior saddle, mid-posterior saddle, or central saddle region. Notably, a wrist-based grip (i.e., gripping the saddle via the wrist) elevates the body’s center of gravity (COG), expands the range of motion (ROM) during leg elevation, and thereby enhances push-off efficiency.

#### 4.2.2 Characteristics of driving stage and their training implications

During the driving phase, athletes employ their lower limbs to propel the sled. Specifically, the rear leg is extended to push off the starting pedals. Subsequently, in the post-launch swing phase of the movement, athletes actively swing their lower limbs while maintaining knee flexion, and thereafter draw their knees toward the torso to generate greater impulse. The lower limbs contact the ground posterior to the hip joints, with body weight borne by the forefeet. The supporting leg is maintained in full extension and aligned with the trunk and head, a configuration that enhances power transmission efficiency during lower limb extension. Athletes should maintain ankle stability and avoid excessive ankle dorsiflexion (i.e., over-cushioning), as this serves to shorten the foot-ground contact time and thereby optimize the resultant ground reaction force. Moreover, athletes are advised to maintain moderate trunk tension and ensure appropriate angular alignment between the trunk and hip joints, as well as between the trunk and shoulder girdle, a factor critical to preserving postural integrity and trunk stability. Regarding arm movements, athletes should actively stabilize their arms at the shoulder joints to keep the sled anterior relative to the body; they should also fully extend their arms to maximize the range of motion of the lower extremities. Further, with the shoulder joints as the pivot, athletes ought to perform controlled anteroposterior arm swings.

The results of this study indicate that athletes can gain a starting advantage by increasing the magnitude of horizontal force and horizontal impulse during the sled push-off phase. Furthermore, anteroposterior stance width (i.e., the distance between the front and rear feet) has no significant impact on the starting advantage, whereas an athlete’s lower limb maximum strength and explosive power are key determinants of such an advantage. During speed training, the squat start technique aligns with the gait characteristics of the special push-off movement. This technique obviates the need for athletes to adjust their anteroposterior stance width (i.e., the distance between the front and rear feet); instead, it focuses on enhancing the amplitude of athletes’ swing legs during the sled push-off phase.

Some research has confirmed the sprint start performance is determined by athletes’ foot contact time and absolute force (Wen, 1994), while a sufficient horizontal force component is essential for achieving horizontal acceleration during the sprint start (Lu, 2010). It has been concluded that athletes should enhance the maximum strength and explosive power of their lower limbs, this improvement can increase the horizontal force and horizontal impulse generated during the sled push-off phase, thereby securing an advantage in the driving phase.

#### 4.2.3 Characteristics of transition stage and their training implications

The transition phase aligns with the acceleration phase of the sled following launch. During this phase, the athlete assumes a position slightly closer to the sled with modest torso flexion, while the supporting leg transitions from backward-oriented to vertical-oriented propulsion. The athlete is required to maintain an appropriate knee flexion angle, expand the range of knee elevation, and simultaneously increase stride frequency and step length, all while sustaining hip height and trunk stability. Furthermore, the athlete must accelerate the sled to maximum velocity during this post-launch phase.

The contact time between the athlete’s supporting foot and the ice surface is exceedingly short; meanwhile, a substantial reaction force is generated at the moment of the supporting foot’s touchdown. Athletes should place equal emphasis on developing greater lower limb power and higher movement velocity. Additionally, optimizing the swing rhythm of the lower limbs and reducing ground contact cushioning duration are key training objectives. Moreover, the study results indicate that during post-launch gait, unilateral sled grip (i.e., single-arm hold) constrains the range of motion (ROM) of the ipsilateral lower limb, leading to asymmetry in step length and running rhythm between the two lower limbs. To address this, athletes should prevent the swinging of the unsupported arm from dissipating propulsive force, avoid hip depression (which reduces lower limb ROM), and refrain from excessive leg extension (to prevent overstriding and subsequent mechanical braking). Furthermore, during the sled’s post-launch period, athletes must maintain an appropriate body-sled distance to avoid dragging the sled. Consequently, strong anti-rotational core strength and trunk stability are required to sustain proper body posture, which in turn enhances power transmission efficiency. Specifically, athletes should avoid excessive trunk flexion caused by insufficient core support and prevent lateral displacement of the body’s center of gravity, thereby avoiding sled deviation from the grooves. Collectively, these measures enable athletes to gain an acceleration advantage.

#### 4.2.4 Characteristics of maximum speed and its maintenance and their training implications

During the maximum speed maintenance stage, athletes achieve and sustain their maximum pushing speeds. During this stage, they also exhibit the longest stride lengths, highest stride frequencies, and shortest ground contact durations. Existing research has indicated that the rational matching of stride length and stride frequency is a key factor influencing push-off speed. Specifically, lower limb extension force exerts a significant effect on stride length (Sun et al., 2010). With precise and dynamic neuromuscular control, athletes should utilize their hip extensor muscles accurately to enhance lower limb driving force (Zheng et al., 2022). Additionally, movement patterns and ground contact duration are two critical factors regulating stride frequency (Sun et al., 2010).

The proper ‘buttock-whipping’ running movement can reduce horizontal braking force during the maximum speed phase, facilitate the development of neuromuscular reflexes, and promote the accumulation of elastic potential energy. Accordingly, maximum speed training can incorporate sprint training methodologies, with an emphasis on facilitating the transformation of physical capacity to adapt to sport-specific movement patterns.

#### 4.2.5 Characteristics of jumping-onto sled and their training implications

The specific technique of sled-mounting via jumping requires athletes to leap forward with one leg and then land chest-first on the sled. Specifically, at the moment of the final ground contact during the sled’s post-launch phase, athletes propel their bodies forward in a jumping motion. They then quickly switch the hand used to grip the sled to the middle of the sled saddle on the opposite side, while the swinging hand also grips the middle of the sled saddle. With their arms maintained in a flexed state, athletes complete the chest-first landing on the sled.

This study demonstrates that athletes exhibit distinct patterns of force application and jumping angles during the post-sled launch phase and the sled-mounting phase. Given that male versus female athletes exhibit differences in body mass and muscular strength. Correspondingly, their plantar pressure during the sled push-off phase is higher than that during the sled-mounting phase. This observation suggests that athletes utilize the inertial momentum of the sled (generated during the post-launch phase) when mounting the sled.

Furthermore, this study demonstrates that no significant difference in plantar pressure was observed between male and female athletes during the sled post-launch phase, indicating that the ground contact force of athletes remains relatively consistent during this phase. Moreover, athletes’ gait characteristics and plantar pressure exhibit no significant alterations, which implies that athletes do not need to execute a specific jumping maneuver in their final step prior to sled landing.

## 5 Conclusion

### 5.1 Technical characteristics of skeleton start

The skeleton start technique can be described as a hybrid of ‘sprint start acceleration running and diving take-off processes’, and is characterized as a speed-power-based periodic movement. Research findings indicate that athletes’ gender and physical capacity exert a significant influence on sled running-off speed, yet exert no significant effect on stride lengths. Furthermore, the anterior-posterior foot stance width of athletes has no notable impact on power transmission efficiency during the sled pushing-off stage.

From the perspective of technical implementation, athletes need to adopt a longer stride after pushing off the sled pedal. During the sled running-off phase, they exhibit an asymmetric periodic running pattern. In terms of force characteristics, the force generated during the sled-jumping phase is smaller than that during the sled running-off phase.

### 5.2 Training implications of skeleton start

For strength training, enhancing core and pillar stability improves athletes’ core/trunk stability in specific postures. Enhancing lower limb maximum strength and explosive power boosts skeleton start performance. Notably, male and female athletes require differentiated strength training protocols for sled pushing-off. The single-leg extension is non-critical for sled-mounting (jumping onto the sled), as athletes do not rely on individual lower limb explosive power during this process. However, sustained explosive power of the individual lower limb is essential for sled running-off.

For speed training, conventional sprint techniques do not fully align with the specialized gait requirements of sled running-off. Thus, a combination of downhill running and sport-specific simulation training is imperative for this phase. Athletes should regularly perform accelerated running exercises to enhance speed, alongside specialized technical simulation drills. Additionally, during downhill and accelerated running, athletes need not adjust their final stride lengths (i.e., no jump is required in the final step).

### 5.3 Limitation

#### 5.3.1 Sample size

In sports biomechanics research, the representativeness of samples is one of the factors determining the external validity of research results. However, the sampling approach adopted in this study, where all participants are from the same national team, exhibits some limitations.

From the perspective of sample attributes, athletes from the same national team typically adhere to unified selection criteria (e.g., body morphology, physiological function thresholds), training systems (e.g., technical movement standards, load distribution patterns), and competitive objectives (e.g., tactical adaptation for specific competitions). This leads to a high degree of homogeneity in the key biomechanical characteristics of the sample. For instance, core indicators such as athletes’ movement force generation patterns, joint movement angle ranges, and muscle activation timing may show convergence due to long-term unified training. Consequently, the sample fails to cover differences in biomechanical characteristics across populations with varying competitive levels, different training systems, or diverse sports foundations.

Future studies should expand the sample source (e.g., including athletes at different levels and with different training backgrounds) and increase sample heterogeneity to enhance the general applicability and practical guiding value of the results.

#### 5.3.2 Discontinuity in research cycles and data acquisition

On-ice training and competitions for skeleton racing are concentrated within a winter window, with a time span of merely 4–5 months. Sports biomechanics research typically requires tracking athletes through a complete cycle, ranging from basic training and specialized intensive training to competitive performance, so as to analyze the dynamic optimization process of technical movements. However, seasonal interruptions make it impossible for researchers to obtain continuous biomechanical data from the off-season to the on-season. Meanwhile, within this short window, athletes must prioritize competition and training tasks, resulting in limited time available for experimental testing, which further restricts the breadth and depth of data collection.

## Data Availability

The raw data supporting the conclusions of this article will be made available by the authors, without undue reservation.
